# The concealed information test with a continuously moving stimulus

**DOI:** 10.1111/psyp.14714

**Published:** 2024-11-01

**Authors:** Lianne N. Wolsink, Ewout H. Meijer, Fren T. Y. Smulders, Robin Orthey

**Affiliations:** ^1^ Department of Cognitive Psychology, Institute of Cognitive Neuroscience, Faculty of Psychology Ruhr University Bochum Bochum Germany; ^2^ Department of Clinical Psychological Science, Faculty of Psychology and Neuroscience Maastricht University Maastricht The Netherlands; ^3^ Department of Cognitive Neuroscience, Faculty of Psychology and Neuroscience Maastricht University Maastricht The Netherlands; ^4^ Aoyama Gakuin University Tokyo Japan

**Keywords:** anticipation, Concealed Information Test, respiration line length, searching concealed information test, skin conductance

## Abstract

The Concealed Information Test (CIT) aims to extract concealed crime‐related knowledge using physiological measures. In the present study, we propose a new variant of the CIT that contains a continuously moving stimulus. A total of 81 participants were either informed or not about the specific location of an upcoming terrorist attack. The CIT consisted of a map with a superimposed moving dot, combined with measurements of respiration and electrodermal activity. The results revealed both respiratory suppression and an increase in skin conductance when the moving dot passed the target location only in informed participants. These findings showed that this new variant of the CIT can differentiate between groups of informed and uninformed individuals and an exploratory analysis revealed it can help narrow down a search area.

## Abstract 2

The multiple‐choice format of the Concealed Information Test (CIT) poses serious restrictions on the information that can be detected. We introduce a new variant of the CIT with a continuously moving stimulus that can differentiate between groups of informed and uninformed individuals and help find a location. This CIT may contribute to improving information gathering by investigative authorities, including the location of explosives, a planned ambush, or where hostages are being held.

## 1 INTRODUCTION

The Concealed Information Test (CIT; Lykken, 1959, 1960) aims to detect concealed crime‐related knowledge by presenting multiple‐choice format questions with one correct answer alternative and several incorrect answer alternatives. For example, “Was the murder weapon a gun? a knife? a rope? a hammer? an ice pick?” The rationale behind the CIT is that only the perpetrator is aware of such intimate details of a crime. Whereas for innocent individuals the answer alternatives are all equally probable, for a guilty individual the correct answer stands out, eliciting a distinct response pattern (klein Selle & Ben‐Shakhar, 2023; Verschuere et al., 2004) resulting in a larger skin conductance response, respiratory suppression, and a larger heart rate deceleration (Meijer et al., 2014).

Although considered a valid procedure for the detection of concealed information, the CIT's practical application is not widespread, with Japan serving as the only exception (Osugi, 2011). One of the reasons for the limited application is that in many cases it can be difficult to come up with a sufficient number of CIT questions about details only the perpetrator knows (Podlesny, 2003). Moreover, the multiple‐choice format of the CIT poses restrictions on what information can be probed, as each question requires several plausible alternatives, excluding, for example, questions with binary yes/no answers (e.g., was the perpetrator wearing a mask?).

To circumvent the limitations posed by discrete answer options, we propose a new variant of the CIT that uses a continuously moving stimulus instead of discrete answer options. Specifically, we familiarized half of our participants with the location of a terrorist attack. The modified CIT displayed a map with a superimposed moving dot. This dot moved along a road, and we expected differences in physiological responses between knowledgeable and unknowledgeable participants at the time the dot moved across the location of the attack.

Specifically, we base this expectation on the work of the role of anticipation on skin conductance and orienting (Spinks et al., 1985; Spinks & Siddle, 1985). In these studies, participants were presented with an imperative stimulus (two‐ or four‐letter matrix in Spinks & Siddle, 1985; one or six letters in Spinks et al., 1985) that they had to identify. A warning stimulus preceded this imperative stimulus and contained information about the imperative stimulus, for example a short message indicating the number of letters and length of presentation (e.g., “2 letter short”; Spinks & Siddle, 1985). Not only was the skin conductance response larger for a more complex imperative stimulus compared with a less complex stimulus, but skin conductance also differed during the interval between warning and imperative stimulus. That is, skin conductance was larger when the warning stimulus signaled a more complex imperative stimulus (Spinks et al., 1985; Spinks & Siddle, 1985). This increase in skin conductance was thought to reflect an active preparation for the processing of subsequent information (Spinks et al., 1985; Spinks & Siddle, 1985), and thus sensitive to anticipation. Anticipation may also be detectable in respiration (e.g., Switzer, 1934). We therefore expected an increasing difference in skin conductance and respiration between informed and uninformed participants when the dot moves across the concealed location.

Besides looking at the difference between knowledgeable and unknowledgeable participants, we also performed an exploratory Searching CIT (SCIT) analysis. Rather than trying to establish guilt or innocence, the SCIT aims to reveal information that the examiner is not aware of (Osugi, 2011). Indeed, in experimental studies, the SCIT has shown to be effective in extracting information from individuals and groups of informed individuals (Breska et al., 2014; Elaad, 2016; Koller et al., 2020; Meijer et al., 2010, 2013; Meixner & Rosenfeld, 2011). Although it is possible to include questions about a location in the SCIT (e.g., Meijer et al., 2010, 2013; see also Osugi, 2011), the discrete answer option format requires at least some prior knowledge to reduce the number of alternatives. Our modified CIT circumvents this problem, and we tested whether the location of the attack could be revealed from the physiological data.

## 2 METHODS

### 2.1 Participants

We tested 81 participants (17 men; age: *M* = 23.46 years, *SD* = 5.61), of which the data of 79 participants were eligible for analysis (see Physiological data processing). As this is a first exploratory study, we did not perform a sample size calculation, and based our sample size on what is reasonable in the CIT field (cf., Lakens, 2022). Participants were mostly undergraduates at Maastricht University. Master students in Legal and Forensic Psychology were excluded from participation, because of their knowledge of concealed information detection. We had no further exclusion criteria. Participation was voluntary and participants signed informed consent before participating in the study. They received an incentive (course credits or €7.50) for participation. The test protocol was approved by the ethics committee of the Faculty of Psychology and Neuroscience, Maastricht University.

### 2.2 Procedure and task

The test session started with the participant reading the study information letter and signing the informed consent. The mock scenario started with the experimenter instructing the participant to imagine being a member of a terror organization. Participants were randomly assigned to either the informed or uninformed condition. In both conditions, the participant received a file containing a map with a blue‐colored road from Kabul to Islamabad, used as an example of a road in this study, and they were instructed to carefully read and memorize the file (for the verbatim instructions see Appendix S2). In the informed condition, this map showed a red dot on the road indicating the exact location of a fictitious roadside bomb planned by the fictitious organization (Appendix S3). The map presented to participants in the uninformed condition did not contain the exact location of the bomb (Appendix S4). We determined the location of the roadside bomb by asking 30 undergraduate students from Maastricht University to indicate the most probable location on the map for a bomb. One recognizable but non‐probable location was chosen as the location of the roadside bomb to minimize false positive physiological responses of uninformed individuals.

After reading the file, experimenter 1 told the participant that they were going to be subjected to several tests by another experimenter, and that their task was to convince the other experimenter that they were unaware of any details from the file, even if that would involve lying. They were promised an extra €2.50 reward if they were successful. For ethical reasons, all participants eventually received the extra reward, regardless of their test performance. Experimenter 1 also told the participant that the other experimenter would not know that these instructions were given to the participant. Experimenter 1 left the room.

Experimenter 2, being blind to the condition of the participant, entered the room and explained to the participant that they were accused of being a member of a terror organization. Experimenter 2 attached the skin conductance sensors and the respiration sensor. The participant was instructed not to move excessively during the tests, to keep an upright posture, and to directly face the screen. Moreover, the participant was made aware that they were monitored through a camera and microphone. The experimenter told the participant that there was reason to believe that the participant had knowledge about an upcoming attack and that the experimenter wanted to find out where the attack would take place by conducting a test. The experimenter gave the participant the task instructions (Appendix S2), where after the participant performed the CIT.

The CIT was programmed using Presentation® software (Version 20.0, Neurobehavioral Systems, Inc., Berkeley, CA, http://www.neurobs.com) and showed the map (Appendices S3 and S4) and the red dot that moved from left to right along the road. The presentation of the map was preceded by instructions, presented in white on a black background for 54 s, to follow the moving dot with the eyes and to not move excessively during the presentation of the map (for task instructions see Appendix S1). Then, the map was presented covering the full width of the screen. The red dot remained static at point A for 16 s, after which the dot started moving across the road from A to B in 110 s. The route contained 1650 *x*‐ and *y*‐coordinates that were each presented for 66.67 ms. Arrived at point B, the dot again remained static for 16 s. The total duration of the trial was 142 s. This trial was repeated four times, resulting in a total duration of the experiment of approximately 10 min. The task is openly available on OSF (https://osf.io/dktcf/).

Afterwards, experimenter 2 disconnected the participant from the sensors and left the room. Experimenter 1 returned. Participants in the informed condition filled in a memory check, whereas participants in the uninformed condition filled in a plausibility check for the location of the bomb. The participant was explicitly instructed to answer the questions honestly and not to hide any information. Lastly, the participant was debriefed.

### 2.3 Physiological data recording

Physiological data were recorded with a 1000 Hz sampling rate using Brain Vision amplifiers (Version 2.1, BrainVision LLC, Morrisville, NC, http://www.brainvision.com). Respiration was recorded by a respiratory band (Disposable Respiratory Effort Belt, Medcat, The Netherlands) positioned around the abdominal or thoracic area, depending on the quality of the data. Skin conductance was recorded by two Ag/AgCl electrodes around the middle phalange of the middle and index finger of the non‐dominant hand. Electrodes were filled with isotonic electrode paste (0.5% NaCl, TD‐246, Medcat, The Netherlands).

### 2.4 Physiological data processing

The raw data were processed using MATLAB (Version 2021b, The MathWorks Inc., 2021). The data were downsampled from 1000 to 10 Hz, resulting in 1420 data points per trial. Skin conductance data were *z* score transformed per trial, relative to the mean and the standard deviation of all data points in that trial. The outcome measure of respiration is respiration line length (RLL; Timm, 1982), which captures both the amplitude and rate of the respiratory response. We computed RLL using the weighted average method (Matsuda & Ogawa, 2011).^1^ We made two changes to the procedure to improve the automatic detection of respiratory cycles and to avoid the need for manual adjustments. First, we applied a 2‐iteration 3‐point moving average filter to the raw data. Second, we removed low‐frequency noise by fitting and subtracting a third‐order polynomial on the data for each trial separately. The next steps were similar to the procedure described in Matsuda and Ogawa (2011), except that we applied a 3 s integration interval instead of a 15 s interval to prevent overly smoothing of our continuous data. In contrast to a typical CIT—where each trial has only one weighted average RLL value for analysis—we were interested in (potentially brief) changes in RLL over time. Using a moving average filter of 15 s would therefore smooth our data too much, thereby suppressing meaningful changes in RLL that are of short duration. We based the decision for a 3 s interval on visual inspection of RLL data and results of the SCIT analysis (see “Exploratory SCIT analysis” section), which relies on accurate peak detection, after applying several time windows for the integration interval.^2^ This procedure led to a total of 1420 moving average samples. Subsequently, *z* scores were calculated per trial, relative to the mean and the standard deviation of all data points in that trial.

Trials were excluded if the response was considered an outlier; a *z* score exceeding 5 or −5. For skin conductance, this led to the removal of data from the fourth trial of one participant in the informed condition.^3^ For respiration, the outlier criterion led to the exclusion of normal respiratory data, as confirmed by visual inspection. As the aim of these outlier criteria is to exclude noisy data that do not reflect true respiration, we adjusted the outlier criterion to 7 and −7. Consequently, for respiration, no outliers were removed. In addition, we excluded the data of two participants because of technical problems and/or excessive movements during the test, leaving a total of 79 participants (39 informed, 40 uninformed).

### 2.5 Data‐analysis

Data analysis was done using MATLAB (Version 2021b, The MathWorks Inc., 2021). We analyzed both the skin conductance and the respiration data using 1420 independent samples *t* tests, one for each sample, with the standardized values as dependent variables and group (uninformed/ informed) as independent variable. To correct for multiple testing, we applied the spatial extent method (Worsley et al., 1992) by only interpreting the results if at least 10 consecutive samples were significant. Because skin conductance, and to a lesser extent respiration, may habituate over trial repetitions (e.g., klein Selle et al., 2018), we executed these *t* tests four times: for the first trial, for the mean of the first and second trial, for the mean of the first, second, and third trial, and for the mean of all four trials. Cohen's *d* values were calculated as measure of effect size (Lakens, 2013). The Cohen's *d* values for RLL were multiplied by −1 to be more easily comparable to the effect sizes for skin conductance. In addition, we calculated the area under the ROC curve (AUC) for each data point (e.g., National Research Council, 2003).

### 2.6 Exploratory SCIT analysis

As an exploratory analysis, we determined for each participant the location of the peak in skin conductance and RLL, and looked at to what extent they could be used at an individual level to narrow down the part of the route to be searched for the bomb. For this, the data processing was adapted. We first applied a low pass filter (2‐iteration symmetrical moving average across 25 data points) over the skin conductance data. Trials were shortened to start with data point 260, to exclude the orienting response due to the start of the movement of the dot. Trials ended at data point 1260 to exclude the 160 data points during which the dot remained static. Skin conductance data were linear detrended. For each participant, we defined the peak as the largest local maximum for skin conductance and the largest local minimum (most negative) for RLL. To quantify the extent to which the peaks were “close to” the target location, we defined for each participant an area that was symmetrical around the participant's peak, and determined whether the area encompassed the target location. In practice, the target would be found if that area around that peak would be searched. The first area had a length of 5% of the total (shortened) trial time. The lengths of the other areas were multiples of the first, up to 100%. For each size of area, we calculated the proportion of participants that had the target encompassed within the area around each individual peak. Without valid data, this proportion of participants with the target in an area will be equal to the proportion of the total area searched, and this is considered “chance performance.” We calculated for each size of area, the smallest proportion of participants within a window that would significantly (*⍺* = .05, two‐sided) deviate from chance level. For informed and uninformed groups separately, we performed this procedure for the first trial, for the mean of the first and second trial, for the mean of the first, second, and third trial, and for the mean of all four trials.

## 3 RESULTS

**FIGURE 1:**
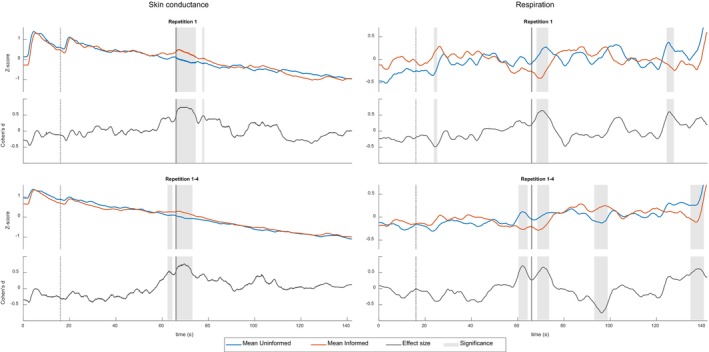
Overview of mean standardized skin conductance (left panel) and respiration (RLL; right panel) per group, and corresponding effect sizes over time. Dashed gray line: start of the movement of the dot; Solid gray line: location of the bomb. The light gray area indicates significant (*⍺* = .05) differences between the groups for these time periods. The graphs show the data of repetition 1 and the average of all four repetitions (repetition 1–4).

Figure 1 shows the mean skin conductance data (left panel) and mean respiration data (right panel) from repetition 1 (upper graphs) and the average of repetitions 1 to 4 (lower graphs) as well as the corresponding effect sizes (Cohen's *d*) and the significance level of the differences.

### 3.1 Skin conductance

Visual inspection of Figure 1 reveals that both the start of the trial and the start of the movement of the dot led to skin conductance responses for both informed and uninformed participants. Participants in the informed group showed a significant increase in skin conductance compared with the participants in the uninformed group at two time intervals. First, when the dot moved over the target location. The significant increase started at 0 s (repetition 1), −0.1 s (average repetition 1–2), −0.9 s (average repetition 1–3) and −0.4 s (average repetition 1–4) relative to the target location. The maximum effect sizes for this effect were *d* = 0.75 (at 4.3 s), 0.74 (at 4.0 s), 0.71 (at 4.3 s), and 0.77 (at 3.5 s) for the first repetition and the averages over the repetitions, respectively, with time points relative to the target location. The maximum AUC values for this effect were 0.69 (at 2.7 and 4.5 s), 0.72 (at 1.9 s), 0.70 (at 0.3 s), 0.69 (at 0.8 s).

Second, there was a significant difference between informed and uninformed participants in the time interval preceding the target location, when averaging over three or all four repetitions. This difference started at −3.6 and −3.7 s relative to the target location, respectively. The maximum effect sizes for this effect were *d* = 0.51 (at −2.6 s) for the average of repetition 1–3 and *d* = 0.55 (at −2.9 s) for the average of repetition 1–4. The maximum AUC values were 0.64 (at −2.8 s) and 0.64 (at −3.4 and −0.2 s). This difference indicates that anticipation also played a role.

### 3.2 Respiration

The respiration data also showed significant differences between informed and uninformed participants. Informed participants showed a significant decrease in RLL when the dot moved over the target location compared with uninformed participants. The significant decrease started at 2.1 s (repetition 1), −4.4 s (average repetition 1–2), −5.3 s (average repetition 1–3), and −5.6 s (average repetition 1–4) relative to the target location. The maximum effect sizes were *d* = 0.65 (at 4.5 s), 0.62 (at 4.5 s), 0.62 (at 4.5 s), and 0.71 (at −3.8 s) for the first repetition and the averages over the repetitions, respectively, with time points relative to the target location. The maximum AUC values for this effect were 0.69 (at 4.7 s), 0.68 (at 4.7 s), 0.69 (at 4.6 s), and 0.69 (at −4.0 and 5.1 s). This difference is followed by an increased RLL that was significant only when averaging over multiple repetitions, likely reflecting a compensatory mechanism (repetition 1–2, minimum *d* = −0.48, minimum AUC = 0.34; repetition 1–3, minimum *d* = −0.69, minimum AUC = 0.29; repetition 1–4, minimum *d* = −0.76, and minimum AUC = 0.28). This significant increase started at 29.7 s, 27.8 s, and 27.1 s after the target location and reached the minimum effect size 30.2 s (for AUC: 30.6 s), 30.2 s (for AUC: 30.3 s) and 30.3 s (for AUC: 30.2 s) after the dot passed the target location, respectively. Moreover, RLL is also significantly increased in the uninformed group toward the end of the trial (repetition 1, maximum *d* = 0.61, maximum AUC = 0.67; repetition 1–2, maximum *d* = 0.58, maximum AUC = 0.66; repetition 1–3, maximum *d* = 0.70, maximum AUC = 0.70; repetition 1–4, maximum *d* = 0.62, maximum AUC = 0.69). For this effect, the maximum effect size occurred 59.5 s (for AUC: 60.4 s), 73.3 s (for AUC: 59.6 s), 72.3 s (for AUC: 72.5 s), and 72.3 s (for AUC: 72.7 s) after the dot passed the target location, respectively.

### 3.3 Exploratory SCIT analysis

**FIGURE 2:**
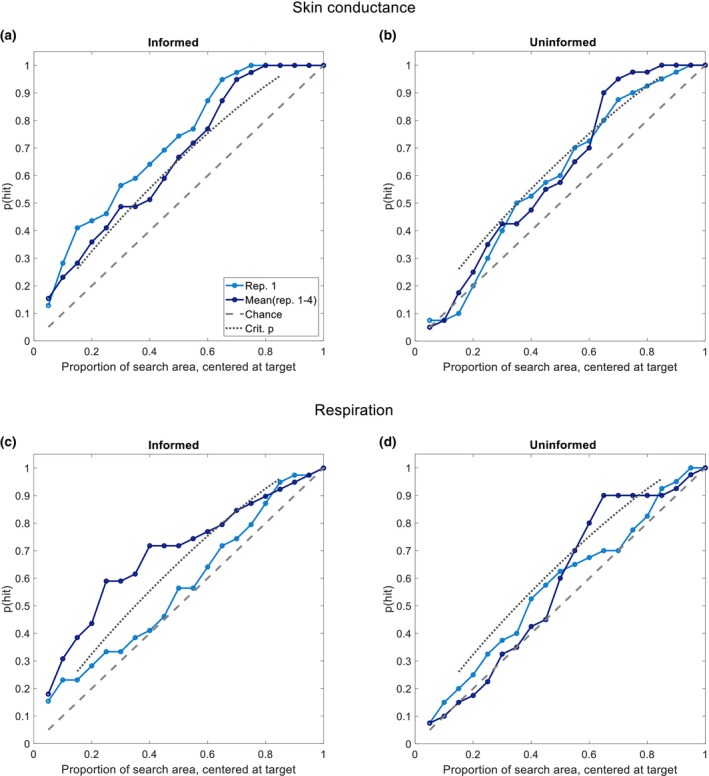
Proportion‐of‐participants against proportion‐of‐road length, given the measurement of peak at some location for a single participant, for skin conductance (upper panel) and respiration (lower panel), for informed and uninformed participants. *p*(hit): proportion of peaks in the time window; Rep. 1: repetition 1; Mean (rep. 1–4): average of all four repetitions; Chance: proportion of hits on chance level. Crit. p: smallest proportion of peaks within a window that significantly (*⍺* = .05) differs from chance level.

Figure 2 shows the probability *p*(hit) of finding the target, given the location of an individual peak, as a function of the length of the area that is searched by the investigative authorities around that peak. This is depicted for skin conductance (panels A, B) and respiration (panels C, D), and for informed (panels A, C) and uninformed participants (panels B, D), separately. Given the location of a peak in one individual, the plots can also be used to find the proportion of the total road investigative authorities should search in order to find the bomb with some chosen probability. The gray dashed line represents chance level. The gray dotted line represents the smallest proportion of peaks within a window that significantly (*⍺* = .05) differs from chance level for each window. For both skin conductance and respiration, the informed group showed a higher than chance level performance, while the uninformed group performed around (for respiration) or more towards chance level (for skin conductance). For example, the probability of finding the target based on skin conductance in the first repetition is about 45% if a length of only 25% of the total search area surrounding the target location is searched. In other words, investigative authorities have a 45% chance to find the bomb when they search in only 25% of the total road. Comparing the data to the critical significance line shows that the data from the uninformed group generally fall below this line until at least 60% of the road surrounding the peak response has been searched, whereas data from the informed group generally fall on or above this critical significance line, except for the first repetition of respiration that does not seem to differ from the uninformed group.

## 4 DISCUSSION

In the current study, we tested a new variant of the CIT with a continuously moving stimulus. In line with our hypotheses, the informed participants showed an increase in skin conductance and respiratory suppression around the concealed location. Only a single repetition was already sufficient to display this effect. The morphology of both skin conductance and respiration indicates that not only anticipation played a role. In fact, the maximum effect size for skin conductance occurred 3.5 s after the dot passed the target location, which is within the time window of a standard skin conductance response (Dawson et al., 2017). Moreover, after passing the target location, the RLL for the informed group increased, likely indicating a compensation response for lower oxygen intake during respiratory suppression.

Effect sizes for our CIT variant were large (skin conductance: maximum *d* = 0.71 to 0.77; respiration: maximum *d* = 0.62 to 0.71), yet smaller than what has been reported for the responses in the normal CIT (skin conductance: d* = 1.55; respiration: d* = 1.11; Meijer et al., 2014). In addition to testing differences between groups, we performed an exploratory SCIT analysis to test if the concealed location could be detected with the physiological responses. The results show that the proportion of peaks surrounding the target location is above chance level for the informed group, whereas the uninformed group performed around chance level (for respiration) or more towards chance level (for skin conductance). Thereby, we were able to narrow down the search area investigative authorities should search for the target location. Future research could also help pinpoint the natural delay of the physiological activity. For example, the latency of the maximum skin conductance difference as expressed in *d* occurred between 3.5 and 4.3 s after the dot moved across the location, a temporal dynamic closely mimicking that of the skin conductance response (Dawson et al., 2017). Fine‐tuning the exact timing would likely result in a more accurate estimate of the target location.

Our results show initial evidence that a continuously moving stimulus can be used in a CIT format. Although the results of this initial study are promising, one important caveat is that the current study was not preregistered. Especially the exploratory SCIT analysis—but also the RLL data processing—contains analytical decisions based on our data. Our findings should therefore be interpreted with caution and before drawing any firm conclusions about the detection efficiency of this new variant of the CIT, the study needs to be replicated. Such future research should also assess the generalizability and practical value of the current findings by using different target locations and different maps, including maps that are familiar to the participants.

In real‐life cases, it may not always be known to the investigative authorities whether a suspect is knowledgeable or not. As a potential solution, several authors proposed a two‐step procedure: where step one determines whether a participant has knowledge or not, and step two then extracts that knowledge (e.g., Breska et al., 2012; Matsuda et al., 2012). These approaches were successfully applied to the normal (S)CIT (Breska et al., 2012, 2014; Matsuda et al., 2009) and could also be suitable for our CIT variant. However, to determine whether a participant has knowledge, the CIT should contain a baseline measure that allows for within‐subject comparisons. In our CIT variant, this could be, for example, a second route without a concealed location.

To conclude, we proposed a new variant of the CIT that contains a continuously moving stimulus, and we presented promising findings for differentiating groups of informed and uninformed individuals, as well as for narrowing down the search area. As such, this new variant of the CIT may contribute to improving information gathering by investigative authorities, including the location of improvised explosive devices, where hostages are being held, or where an ambush is planned.

## AUTHOR CONTRIBUTIONS

**Lianne N. Wolsink:** Data curation; formal analysis; investigation; visualization; writing – original draft. **Ewout H. Meijer:** Conceptualization; formal analysis; methodology; project administration; resources; supervision; visualization; writing – review and editing. **Fren T. Y. Smulders:** Formal analysis; validation; visualization; writing – review and editing. **Robin Orthey:** Software; writing – review and editing.

## CONFLICT OF INTEREST STATEMENT

The authors declare no conflicts of interest.

## Supporting information


**Appendix S1.** Task instructions
**Appendix S2.** Experimenter instructions
**Appendix S3.** File informed condition
**Appendix S4.** File uninformed condition

## Data Availability

The data that support the findings of this study are openly available on OSF at https://osf.io/dktcf/.

## Footnotes

^1^
We initially calculated RLL using the method described by Timm (1982) and applied a moving average filter to the RLL values. Upon reviewers' request, we changed the RLL calculation to better account for changes in respiratory cycle. The methods, results, and code for the initial analysis can be found on OSF (https://osf.io/dktcf/).

^2^
The results of the analyses employing a 0 and 15 s integration interval can be found on OSF (https://osf.io/dktcf/) and can be reproduced using the RLL preprocessing script.

^3^
For another participant in the informed condition, one data point of trial 1 had a physiologically impossibly low (and below −5) value compared with the surrounding data points. We replaced this value through linear interpolation and included this participant's data in the analyses.
