# Supplementary material for: The concealed information test with a continuously moving stimulus
Source: Psychophysiology. 2024 Nov 1;62(2):e14714. doi: 10.1111/psyp.14714 (PMC11870813; doi:10.1111/psyp.14714)

**Appendix S1: Task Instructions**

“You will be presented with a map and a red dot. After a while, the red dot will start moving across the route from Kabul to Islamabad. You will need to follow the moving dot with your eyes. This will be checked using the camera in front of you. Failure to follow the dot with your eyes will be seen as an attempt to sabotage the test, and will forfeit the reward. Also, make sure that you sit still during the time the map is on the screen. Movement will also be seen as an attempt to sabotage the test, and will forfeit the reward.”

**Appendix S2: Experimenter Instructions**

**Experimenter 1:** “Imagine you are a member of a terror organization based in the Middle East. You will receive a file that contains some details about this organization. Your task is to read this file carefully and memorize as many details as possible.”

The participant receives the file and about 3 minutes to read it.

**Experimenter 1:** “Next you will be subjected to a number of tests to determine what kind of information you learned from this file. Another experimenter is conducting these tests and it is your task to beat these tests. In the end the (other) experimenter should think that you were unaware of any details from the file. If you manage to convince the other experimenter that you have no knowledge about this terror organization you will be rewarded with an additional 2.50€ leading up to a total of 10.00€ for participating in this experiment.”

Experimenter 1 leaves and experimenter 2 enters the room.

**Experimenter 2:** “You are accused of being a member of a terror organisation. Next, I will subject you to a number of tests to determine what information about this terror organisation you have. During these tests we will measure your physiological reactions. In particular sweat in the palm of your hands, and breathing patterns.”

Experimenter 2 attaches the skin conductance and respiration sensors.

**Experimenter 2:** “I have reason to believe you have knowledge about an upcoming attack along the route from Kabul to Islamabad. With this test, we want to find out where that attack will take place. You will be presented with a map and a red dot. After a while, the red dot will start moving across the route from Kabul to Islamabad. You will need to follow the moving dot with your eyes. This will be checked using the camera in front of you. Failure to follow the dot with your eyes will be seen as an attempt to sabotage the test, and will forfeit the reward. Also, make sure you sit still during the time the map is on the screen. Movement will also be seen as an attempt to sabotage the test, and will forfeit the reward.”

The participant performs the CIT.

**Experimenter 2:** “Ok, this is the end of this part.”

Experimenter 2 leaves and experimenter 1 enters the room again.

**Experimenter 1**: “Ok, the main part of the experiment is over. From now on, please answer all questions honestly and do not hide anything.”

Participants fill in the memory or plausibility check, and they are debriefed.

**Appendix S3: File Informed Condition**

This terrorist organisation also planted a road side bomb alongside the blue-coloured road on the map below. The exact location of the bomb is indicated with a red dot.

**
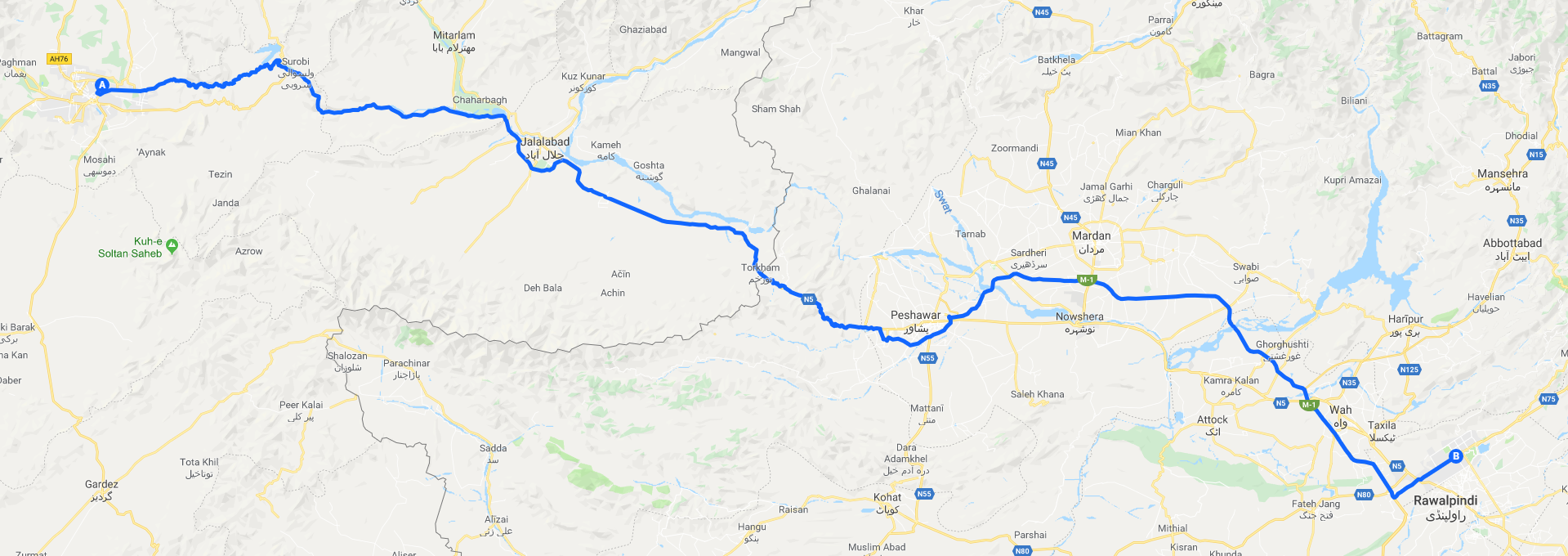
**

**Appendix S4: File Uninformed Condition**

This terrorist organisation also planted a road side bomb alongside the blue-coloured road on the map below.


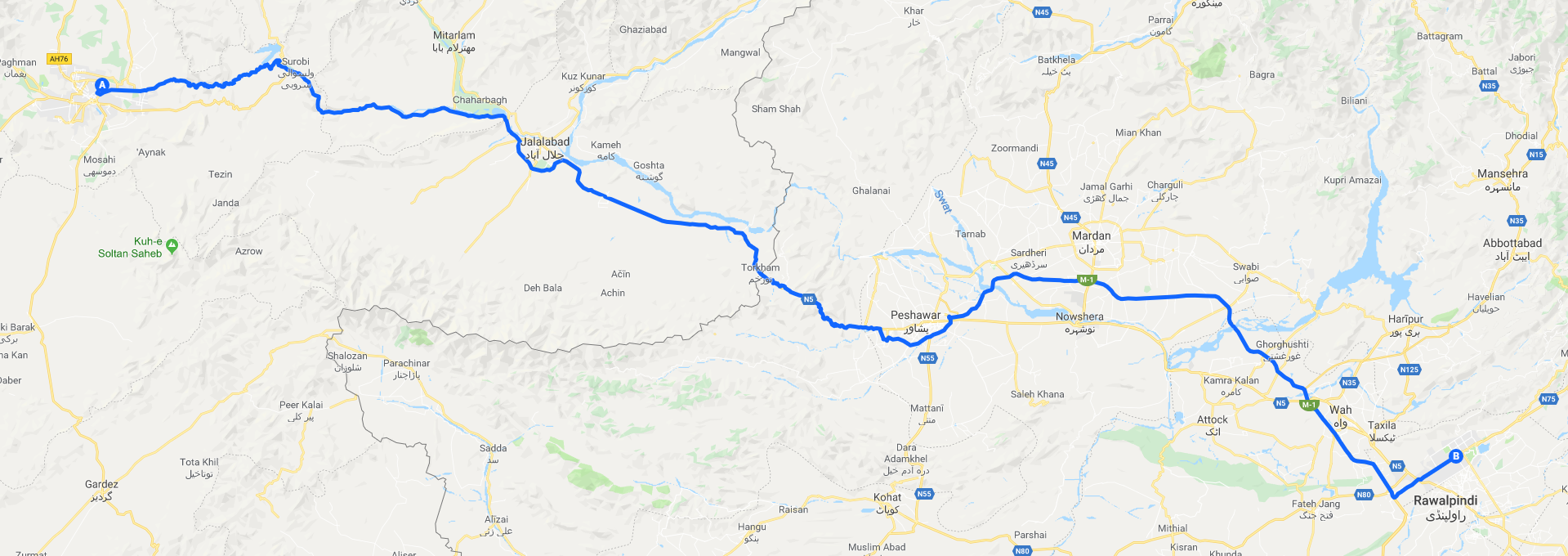

Supplement: Supplementary file 1 — Appendix S1. Task instructions Appendix S2. Experimenter instructions Appendix S3. File informed condition Appendix S4. File uninformed condition [file PSYP-62-e14714-s001.docx]
